# Gray and White Matter Metrics Demonstrate Distinct and Complementary Prediction of Differences in Cognitive Performance in Children: Findings from ABCD (*N* = 11,876)

**DOI:** 10.1523/JNEUROSCI.0465-23.2023

**Published:** 2024-02-22

**Authors:** Lea C. Michel, Ethan M. McCormick, Rogier A. Kievit

**Affiliations:** ^1^Cognitive Neuroscience Department, Radboud University Medical Center, Nijmegen 6525 GA, The Netherlands; ^2^Methodology and Statistics, Institute of Psychology, Leiden University, Leiden 2333 AK, The Netherlands; ^3^Department of Psychology and Neuroscience, University of North Carolina, Chapel Hill, North Carolina 27599-3270

**Keywords:** cognitive performance, gray matter, regularization, structural equation modeling, white matter

## Abstract

Individual differences in cognitive performance in childhood are a key predictor of significant life outcomes such as educational attainment and mental health. Differences in cognitive ability are governed in part by variations in brain structure. However, studies commonly focus on either gray or white matter metrics in humans, leaving open the key question as to whether gray or white matter microstructure plays distinct or complementary roles supporting cognitive performance. To compare the role of gray and white matter in supporting cognitive performance, we used regularized structural equation models to predict cognitive performance with gray and white matter measures. Specifically, we compared how gray matter (volume, cortical thickness, and surface area) and white matter measures (volume, fractional anisotropy, and mean diffusivity) predicted individual differences in cognitive performance. The models were tested in 11,876 children (ABCD Study; 5,680 female, 6,196 male) at 10 years old. We found that gray and white matter metrics bring partly nonoverlapping information to predict cognitive performance. The models with only gray or white matter explained respectively 15.4 and 12.4% of the variance in cognitive performance, while the combined model explained 19.0%. Zooming in, we additionally found that different metrics within gray and white matter had different predictive power and that the tracts/regions that were most predictive of cognitive performance differed across metrics. These results show that studies focusing on a single metric in either gray or white matter to study the link between brain structure and cognitive performance are missing a key part of the equation.

## Significance Statement

This paper enriches the recent debates on the challenges of linking variation in brain structure to phenotypic differences (Marek et al., 2022). By using latent variables (to improve power) and structural equation modeling (to allow greater flexibility in linking brain to behavior), and by simultaneously incorporating multiple measures of gray and white matter in a large sample, we demonstrate relatively strong and robust brain–behavior associations, which highlight the complementarity of gray and white matter metrics in predicting cognitive performance as well as the importance of incorporating the full complexity of these associations over one-to-one linkages. This finding should lead researchers to consider integrating both gray and white matter measures when demonstrating a more comprehensive picture of brain–cognition relationships.

## Introduction

The field of cognitive neuroscience is premised on the hypothesis that differences in cognitive performance can be understood in part by studying differences in brain structure and function (Basten et al., 2015). Although much is known about the relationship between gray and white matter structure and cognitive performance (Tamnes, Østby, Fjell, et al., 2010; Tamnes, Østby, Walhovd, et al., 2010; Magistro et al., 2015; Muetzel et al., 2015; Schnack et al., 2015; Deoni et al., 2016), less is known about how the structure of the two tissues together explain differences in cognitive performance.

One way to look at this challenge is to consider gray and white matter as two sides of the same coin. At the cellular level, they are both composed of neurons, although different parts [cell bodies, dendrites, synapses, and axons for gray matter and (un)myelinated axons for white matter], glial cells, and vasculature (Wandell, 2016; Purves et al., 2019). Moreover, recent findings observe even more overlap, namely, that differences in one tissue (white matter myelination) may affect the other tissue [cortical thickness (CT); Natu et al., 2019], suggesting they may capture the same neurobiological properties using different techniques.

A counterargument would be that gray and white matter are two distinct brain properties with distinct mechanistic roles. A twin study reports that gray and white matter volume shared 68% heritability, suggesting both overlapping and distinct genetic mechanisms (Baaré et al., 2001), and recent attention has focused on their different transcriptome patterns, highlighting the differentiation of their cells and their specific functional roles (Mills et al., 2013). Studies have shown the importance of both dendritic network and myelinated axons to provide faster and more efficient cognitive performance (Kail, 1997; Tamnes et al., 2012; Rolls and Deco, 2015), thus illustrating the potentially complementary roles of gray and white matter to explain differences in cognitive performance.

Beyond the conventional classification of gray and white matter, it is important to consider the heterogeneity within these tissues. Each specific region/tract within gray and white matter may offer unique or shared information in predicting cognitive performance, both within a single metric and across diverse metrics. Understanding the intricate interplay between these regions/tracts and metrics can provide a more comprehensive understanding of brain–behavior associations.

In summary, there is compelling evidence to suggest that gray and white matter play complementary roles explaining differences in cognitive performance. To date, few studies investigated the combining effects of gray and white matter differences on cognitive performance, and so far the literature point toward a complementary roles of the two tissues (Østby et al., 2011; Kievit et al., 2014; Ritchie et al., 2015).

The causes of this paucity are manifold. First, study designs tend to focus on either classical MRI sequences (T1, T2, T2*) or diffusion-weighted imaging (DWI) data. These are sequences with distinct scanner demands and require dedicated analysis expertise, which often leads to papers focusing on one tissue. Moreover, the standard implementation in neuroimaging software, where a brain region/voxel is the outcome of a regression equation, makes it considerably more challenging to implement models where multiple brain metrics predict a single phenotypic outcome (e.g., cognitive performance). Finally, the high number of predictors of such models might hinder their feasibility, decrease the statistical power, and generate false positives.

To overcome these challenges, we investigated the unique predictive roles of gray and white matter metrics in predicting cognitive performance in childhood. By conducting a multimodal analysis in a regularized structural equation model in a large sample, we overcome many existent weaknesses in previous work, and it allowed us to maximize the likelihood of distinguishing the competing hypotheses of interest. In addition, we will explore the regional distribution of these associations within and across metrics, as well as identify which specific metrics in gray and white matter are more influential in predicting cognitive performance.

This study provides new insights into the complementary information provided by gray and white matter in supporting cognitive performance.

## Materials and Methods

### Participants

The ABCD Study (https://abcdstudy.org/) is an ongoing longitudinal study across 21 data acquisition sites enrolling 11,876 children from 9 to 16 years old. For more information on ABCD protocols and inclusion/exclusion criteria, see Volkow et al. (2018).

This paper analyzed the baseline sample (9–11 years old) from release 4.0 (https://abcdstudy.org/; http://doi.org/10.15154/1523041) that includes a sample of 11,876 children. Data entry outliers for 10 participants in the Little Man task were replaced with NA and included in the full information maximum likelihood (FIML) estimation. We included participants with partial data across cognitive tasks and the neuroimaging data in our models.

**Figure 1.**
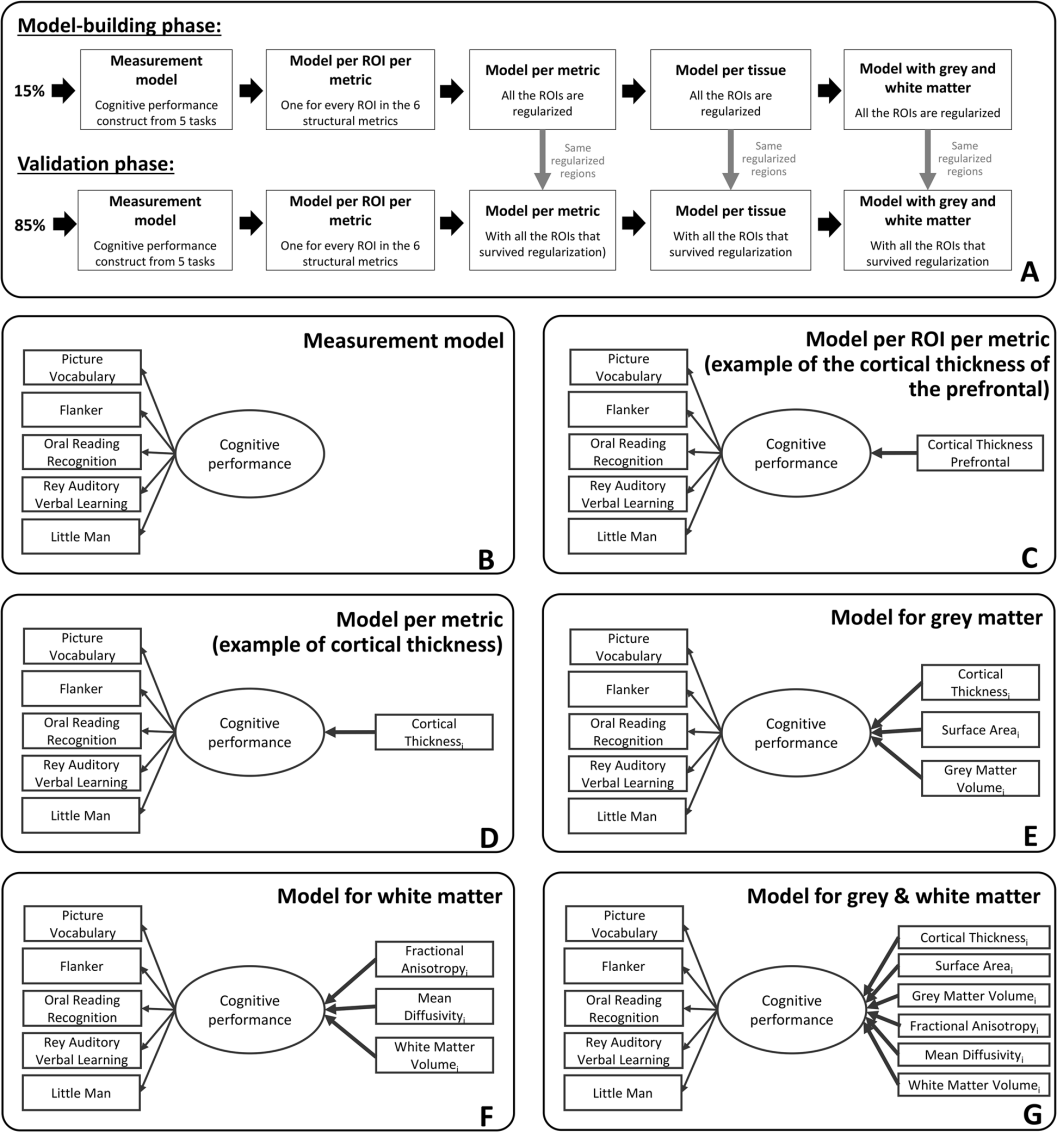
***A***, Steps followed during the model-building and the validation phases of the study. The regions of interest fitted into the models of the validation phases resulted from an initial regularization made in the model-building phase. ***B***, Measurement model of cognitive performance based on five cognitive tasks. ***C***, Example of a MIMIC model path diagram of a model per ROI per metric where one metric of one region of interest (CT of the prefrontal) predicts cognitive performance. ***D***, Example of a MIMIC model path diagram of a model per metric where all regions of interest of one metric (here CT) that survived regularization predict cognitive performance. ***E***, MIMIC model path diagram for the gray matter model with CT, SA, and GMV predicting cognitive performance. *i* represents the regions of interest from each metric in gray matter that survived regularization in the model and can go from 1 to 34 (as we use the Desikan–Killiany atlas and average bilaterally). ***F***, MIMIC model path diagram for the white matter model with FA, MD, and WMV predicting cognitive performance. *j* represents the regions of interest from each metric in white matter that survived regularization in the model and can go from 1 to 20 (as we use the AtlasTrack atlas and average bilaterally). ***G***, Path diagram for the gray and white matter model with CT, SA, GMV, FA, MD, and WMV of the regions that survived regularization predicting cognitive performance.

Given the complexity of the analysis and the a priori challenges associated with high-dimensional regularized structural equation modeling (SEM) approaches, a fully preregistered analysis was not possible. However, to ensure robustness of our findings, we divided the sample into two subsets, allowing us to balance exploratory model optimization and validation in a nonoverlapping sample. In our study, a random sample of 15% of the data was used to optimize the model-building and estimation steps, and the other 85% of the sample was used as a validation sample (Srivastava, 2018; Fig. 1). The set of regions used in the validation sample was derived from the best predictive regions identified in regularized models estimated using the model-building sample (Schwarz, 1978; the regularization techniques will be explained more below, Experimental design and statistical analysis). This strategy allows us to jointly optimize robustness and flexibility in cases where model estimation, adaptation, and convergence are nontrivial while maintaining sufficient power in the validation set to be sufficiently well powered.

The model-building sample consisted of 1,781 children (49.1% female; mean age, 9.9; SD = 0.6; range, 8.9–11.1), and the validation sample included 10,095 children (47.6% female; mean age, 9.9; SD = 0.6; range, 8.9–11).

### Cognitive performance

In our modeling approaches, we want to focus on construct capturing a broad sampling of cognitive ability. To this end, we use the same procedure as identified in Sauce et al. (2022) by selecting five cognitive tasks from the NIH Toolbox Cognition Battery: Picture Vocabulary, Flanker, Oral Reading Recognition, Rey Auditory Verbal Learning, and Little Man (Sauce et al., 2022). They measure respectively language vocabulary knowledge, attention and inhibitory control, reading decoding skill, verbal learning and memory, visuospatial processing flexibility, and attention. All of the tasks were administered using an iPad with support or scoring from a research assistant where needed. For more information on each task, see Luciana et al. (2018).

Next we specified a confirmatory factor model that posits a single latent factor for cognitive performance, reducing measurement error and increasing precision. It enables the model to compute the common variance between these five tasks and thus to have a more accurate representation of cognitive performance (Fig. 1*B*). The measurement model has already been validated in another study; for more information on the rationale for the choice of cognitive tasks and the measurement model, see Sauce et al. (2022). Note that the model chosen here should not be interpreted as a commitment to a strong “causal g” (Kievit et al., 2017), but rather as a way to specify a broad cognitive factor that will maximize our statistical power to capture the patterns of interest. A fruitful avenue of future work will be to examine the degree (or lack of) overlap in the neural mechanisms predicting each individual cognitive domain—a goal beyond the present paper.

### Brain structure measures

MRI and diffusion tensor imaging (DTI) scans were collected across the 21 research sites using Siemens Prisma, GE 750, and Philips 3 T scanners. Scanning protocols were harmonized across sites and scanners. Full details of all the imaging acquisition protocols and the processing methods used in ABCD are outlined elsewhere (Casey et al., 2018; Hagler et al., 2019).

To determine the importance of gray and white matter, we chose six structural metrics available in the ABCD Study: CT, surface area (SA), and volume (GMV) for gray matter and fractional anisotropy (FA), mean diffusivity (MD), and volume (WMV) for white matter.

### Gray matter

Gray matter measures were estimated from MRI scans with FreeSurfer (version 7.1.1).

The processing steps involved cortical surface reconstruction, subcortical segmentation, removal of nonbrain tissue, Talairach transformation, segmentation of white matter and deep gray matter structures, intensity normalization, and surface deformation. The images were registered to a spherical atlas based on individual cortical folding patterns, and the cerebral cortex was parcellated into 34 regions per hemisphere with the Desikan–Killiany atlas (Desikan et al., 2006). The full pipeline can be found elsewhere (Hagler et al., 2019).

The FreeSurfer output includes volume, CT, and SA (Casey et al., 2018). Considering the challenges of dimensionality in our SEM, we decided to average every region of interest bilaterally (i.e., the brain was parcellated in 34 regions across the brain for each metric).

### White matter

White matter structure can be measured through DWI and structural MRI (Wandell, 2016). DWI is a technique that allows researchers to record the diffusion of water in the brain using DTI to model the diffusion within the axons and myelin sheath, thus analyzing the directionality of the white matter tracts (Le Bihan, 2003; Assaf and Pasternak, 2008). Structural MRI provides a measure of volume, while DTI computes FA, MD, radial diffusivity, and axial diffusivity (AD).

In the ABCD Study, diffusion measures were obtained using tabulated diffusion MRI data from a high angular resolution diffusion imaging (HARDI) sequence with multiple *b* values. The images were corrected for head movement, eddy current distortions, B0 distortion, and gradient nonlinearity distortion. The resulting diffusion measures were used for subsequent analyses. The processing steps are described elsewhere (Hagler et al., 2019).

White matter measures include WMV, FA, and MD. The tracts were divided with the AtlasTrack atlas which created 37 regions of interest (17 regions per hemisphere and 3 regions aside) (Hagler et al., 2019). We followed a similar procedure as with the gray matter metrics and averaged every tract of interest bilaterally.

### Experimental design and statistical analysis

Next, we specified the core questions investigated by our analyses.

1. Do the metrics of gray matter and white matter demonstrate complementary roles predicting cognitive performance?
2. Do the different metrics in each tissue have unique predictive roles? If so, which metric is the most important to predict cognitive performance?
3. For each metric, do the different regions of interest have unique predictive roles?
4. Do the same regions of interest have the strongest predictive power across different metrics in each tissue?

Our focus will be on both the tissue scale (gray and white matter) and the metrics scale (CT, SA, GMV, FA, MD, and WMV). For each scale, we will study if the predictors of cognitive performance each have a unique role (i.e., there is no overlap in the information contributed by the different variables to predict cognitive performance) or if they have complementary roles (i.e., there is a partial or total overlap in the information contributed by the different variables to predict cognitive performance).

The study uses structural equation model approach to evaluate the hypothesis that brain structural metrics predict the latent variable, cognitive performance. Structural equation models, and more particularly MIMIC models (multiple-indicator multiple-cause), offer an effective way to model cognition as a latent variable and to estimate the contribution of multiple simultaneous hypothesized causes to explain individual differences in cognitive performance (Jöreskog and Goldberger, 1975; Kievit et al., 2014).

First, we estimated a series of structural equation models to test how each metric in each individual region/tract predicted cognitive performance (i.e., every region in all the six metrics have been computed independently). The key question of interest is the strength of the key parameter highlighted in bold (Fig. 1*C*).

Next, to assess how each metric predicted cognitive performance, we used a regularized SEM approach (Jacobucci et al., 2019) which incorporates a penalty on key parameters of interest. Specifically, it allows us to have many regions/metrics simultaneously predict the outcome, with a penalty on the path estimates from brain metrics to the cognitive latent variable, that induces sparsity. Regularization is a method that imposes a penalty in order to decrease the complexity of the model while keeping the variables that are the most important in predicting the outcome. For instance, it allows us to include the CT measures from all 34 regions of interest as simultaneously predicting cognitive performance with a lasso penalty that pushes parameter estimates of small or absent effects to 0 and retains only those regions that contribute meaningfully in predicting the outcome for the validation sample. This accounts for the partial overlap in variance arising from the inherent way the metrics are measuring the same underlying structure. For instance, GMV is a product of SA and CT. If GMV functions as a deterministic transformation with no additional information beyond CT or SA for cognitive performance, our regularization approach should ensure all redundant parameter estimates are pushed to 0. For example, if the CT, SA, and GMV of the frontal pole each have large effects on cognitive performance and these effects are redundant (they include the same information to predict cognitive performance), then the measures with the smaller effects of the three will be pushed to 0.

We developed a procedure to select a subset of regions/tracts based on their predictive ability in the model-building sample (e.g., 15% of the total sample). The output of each regularized model estimation was a set of all the regions/tracts with a regularized β different from zero, or considered “important” in a regularized framework. These regions were entered into the models predicting cognitive performance in the validation sample (e.g., 85% of the total sample). We implemented this process across six models per metric (Fig. 1*D*), two models per tissue (Fig. 1*E*,*F*), and the model combining gray and white matter metrics (Fig. 1*G*). The benefit of this approach is to have a parsimonious representation of the key regions that help predict cognitive performance in each metric. We also examined “full” models combining every region of interest within a metric (e.g., the 34 regions for CT), within a tissue (e.g., the 102 regions for the three gray matter metrics and the 60 regions for the three white matter metrics), and within gray and white matter metric (e.g., the 162 regions across the six metrics).

To compare the predictive information of gray and white matter, we fitted three models as follows: one model with the regions extracted from the regularization of the three gray matter metrics, one model with the regions extracted from the regularization of the three white matter metrics, and a final model with the regions extracted from the regularization of the gray and white matter metrics (e.g., as displayed in Fig. 1*A*, the regions were selected because they survived the regularization in the model-building sample). For each model, we use a likelihood ratio test to examine whether the inclusion of a tissue (gray/white matter) or a metric within a tissue (e.g., CT) improves the model compared with a model where the paths corresponding to an additional metric/tissue are constrained to 0. For each final model, we extracted the (adjusted) *R*-squared to assess the proportion of variance in cognitive performance explained by each model.

**Figure 2.**
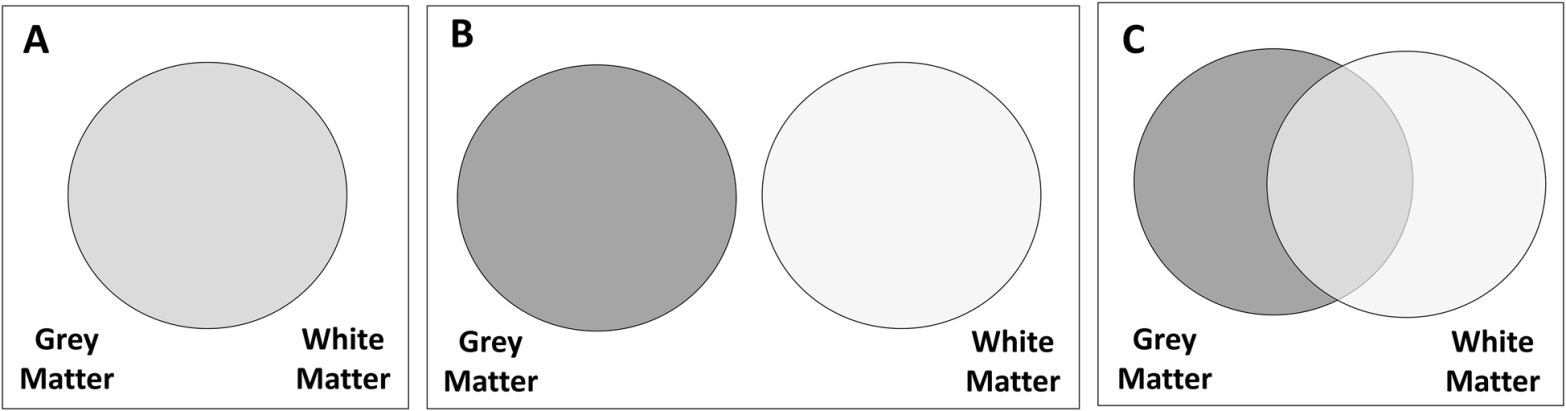
Hypotheses regarding the information provided by gray and white matter metrics. ***A***, Redundant, gray, and white matter give the same information. ***B***, Independent, gray, and white matter give completely different information. ***C***, Partially overlapping, gray, and white matter give both similar and different information.

From the analyses, we can imagine our results being captured by one of three (simplified) scenarios as follows:

1. Gray and white matter metrics give the same, noncomplementary information to predict cognitive performance. The models with only gray matter, only white matter, or both will have a similar adjusted *R*-squared (Fig. 2*A*), and model selection would favor a model with only one tissue.
2. Gray and white matter metrics give fully distinct/complementary information to predict cognitive performance. Under this scenario, model selection would favor a model including both tissues, and the joint *R*-squared would approximate the sum of the *R*-squared of each tissue in isolation (Fig. 2*B*).
3. Gray and white matter metrics give complementary, but partially overlapping, information to predict cognitive performance. In this case, model selection would favor a model with both tissues, and the joint *R*-squared will be higher than the one of the most predictive tissue, but lower than the sum of the *R*-squared of each tissue in isolation (i.e., the *R*-squared are neither interchangeable nor fully additive; Fig. 2*C*).

These analyses were replicated separately for male and female participants to examine the potential impacts of sex differences in brain structure for the findings. In an additional analysis, total intracranial volume (TIV) was added as a third predictor to explain the differences in cognitive performance. Accounting for TIV in the model is a contentious issue (Hyatt et al., 2020), as it modifies the question from whether it is the absolute size (or thickness, anisotropy, etc.) of a region that matters to predict cognitive performance or the relative measure given a participant's TIV.

Regularization has many strengths, as outlined before, but this comes at a specific cost, namely, its attempt to build a simplified model. In other words, if two regions have almost the same predictive power, but are somewhat redundant in this regard, a regularized model is likely to push the parameter estimate of one of the two regions to 0 and “keep” the other region instead. If we were to report the regional pattern as such, this may lead to an overinterpretation of the absence and presence of some regions. To examine the regional specificity of our findings, we conducted a repeated regularization procedure, iterated 1,000 times using a different 15% sample on each occasion. Across these 1,000 iterations, we computed the percentage of times each region survived regularization (i.e., when a given region had a nonzero estimate in the final model). This approach allowed us to assess the stability of each region/tract in providing unique information to explain variations in cognitive performance. Within each model (Fig. 1*D–G*), we calculated the percentage of instances in which a region/tract survived regularization. Regions/tracts with higher percentage values demonstrate a more consistent and distinct contribution to the prediction of cognitive performance. In the model employing a single metric (Fig. 1*D*), this reflects the differential information provided by distinct regions/tracts within the same metric. In the models incorporating multiple metrics (Fig. 1*E–G*), the likelihood of regions/tracts surviving regularization is lower if they provide redundant information across different metrics. Therefore, regions/tracts with a higher percentage not only demonstrate a unique predictive role across different regions/tracts but also across the various metrics.

We used the following guidelines to assess the good fit of the models: root mean square error of approximation (RMSEA) <0.05 (acceptable, 0.05–0.08), comparative fit index (CFI) >0.97 (acceptable, 0.95–0.97), and standardized root mean square residual (SRMR) <0.05 (acceptable, 0.05–0.10; Schermelleh-Engel et al., 2003; Mueller and Hancock, 2008). We compared models’ fit using the Akaike information criterion (AIC) and the Bayesian information criterion (BIC). These parameters penalize models with a higher number of predictors, thus encouraging the selection of more parsimonious models that explain the data well while avoiding overfitting.

All analyses were carried out on the data of the first wave using R, version 4.1.0 (http://www.r-project.org/) and the lavaan package (Rosseel, 2012). All models were fit using maximum likelihood estimation, with FIML to account for missing data and robust estimation with adjusted standard errors to deal with deviations from (multivariate) normality.

### Data and code accessibility

Data can be requested through https://nda.nih.gov/, and the code to reproduce our analyses is available on https://osf.io/ryskf/.

## Results

### Measurement model (validation, parameters, and invariance)

To assess cognitive performance, we used the same measurement model built by Sauce et al. (2022) in a slightly different sample. Model estimates were highly similar.

The confirmatory factor model fitted the data well in the validation sample, *x*^2^ = 156.404; degrees of freedom (df) = 5; *p* < 0.001; RMSEA = 0.055 (0.00–0.070); CFI = 0.980; SRMR = 0.020. This result demonstrates that the common variance among all five cognitive tasks can be captured by one latent variable that we call here “cognitive performance.”

**Figure 3.**
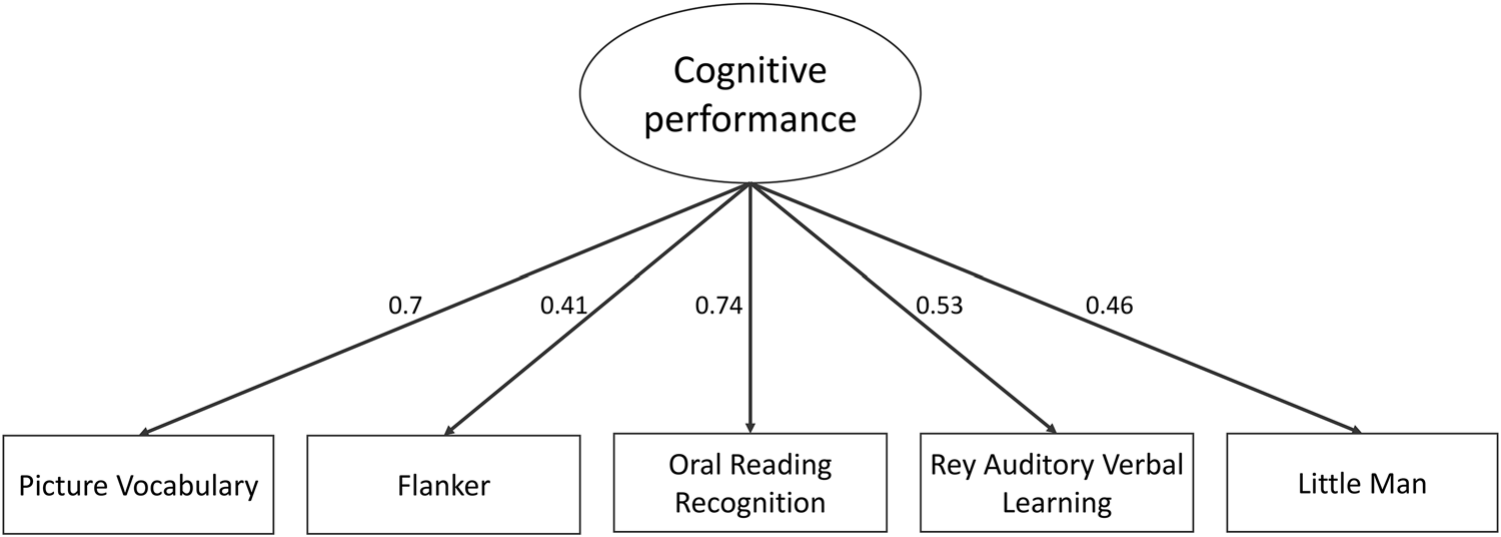
Path diagram of a measurement model of cognitive performance based on five cognitive tasks. All variables are standardized and significant.

Oral Reading Recognition task and Picture Vocabulary task have the strongest standardized factor loadings (0.74 and 0.7, respectively). Rey Auditory Verbal Learning task, Little Man task, and Flanker task are mildly predicted by the construct between 0.41 and 0.53 (Fig. 3). The results show little change when we add age as a predictor of the latent variable.

### Gray and white matter give both overlapping and unique information to predict cognitive performance

We fitted the full model including all regions/tracts that survived regularization in gray and white matter metrics and compared it with a model that includes the same predictors but constrains either all gray or all white matter predictors to 0. This comparison allows us to test if the metrics in gray matter and white matter have complementary roles predicting cognitive performance.

The model with both gray and white matter fitted the data well [*x*^2^ = 793.903; df = 205; *p* < 0.001; RMSEA = 0.017 (0.016–0.019); CFI = 0.935; TLI = 0.918; SRMR = 0.011] and showed the best performance among the three models (AIC_diff_ > 265; BIC_diff_ > 136 in favor of the model with both gray and white matter) and explained 19.0% of the variance in cognitive performance. The model with only gray matter explained 15.4% of the variance and the one with only white matter explained 12.4% of the variance (Table 1).

**Table 1.** Estimates of the models’ fit and the variance explained by the predictors within each model

	Gray matter model	White matter model	Gray and white matter model
*R*^2^ (adjusted)	0.154	0.124	**0**.**190**
AIC	122,941.1	123,153.5	**122,675**.**3**
BIC	123,277.4	123,389.6	**123,140**.**5**

The best model is shown in bold.

These findings demonstrate that gray and white matter bring both overlapping and unique information to predict the differences in cognitive performance in line with hypothesis C—“partially overlapping”—in Figure 2.

These results were replicated in samples comprising exclusively of male (*R*-square adjusted for gray matter and white matter = 17.4%, for gray matter = 14.6%, and for white matter = 10.8%) and also female participants (*R*-square adjusted for gray matter and white matter = 23.0%, for gray matter = 17.8%, and for white matter = 15.9%).

In the model accounting for TIV, we also compared a model with only TIV as a predictor and a model with gray matter, white matter, and TIV as a predictor instead of only gray and white matter. The model with gray matter, white matter, and TIV explained the best variance in cognitive performance (*R*-square adjusted for gray matter and white matter and TIV = 19.1%), while the model with only TIV explained 6.8%. The addition of TIV as a predictor did not improve the fit of the model, nor did its inclusion substantially altered the parameter estimates of the regions/tracts.

**Figure 4.**
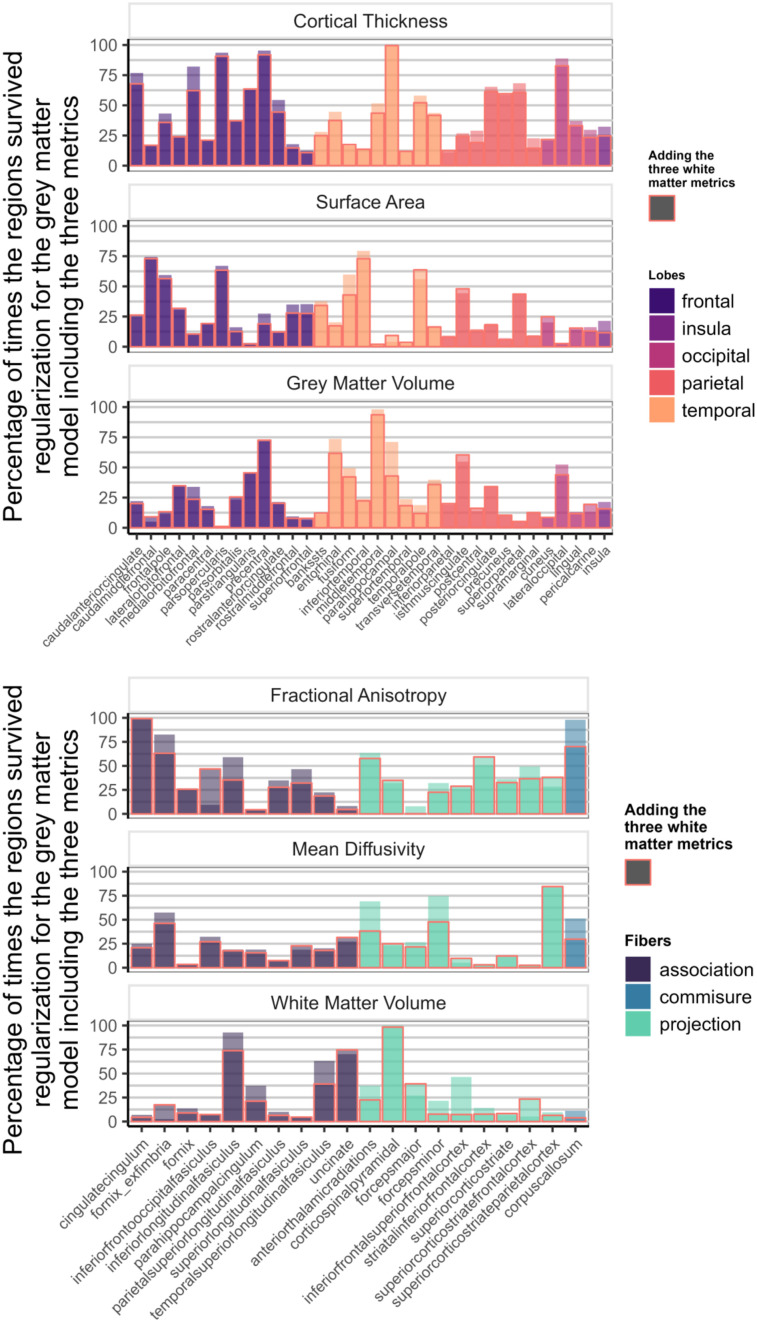
Percentage of times one region/tract in a metric survived regularization in the model with the three metrics of one tissue (top, gray matter; bottom, white matter; Fig. 1*E*,*F*). The columns highlighted in red represent the percentage of survival for the region when the model includes the metrics of the other tissue (Fig. 1*G*).

Figure 4 illustrates the overlap in information within gray and white matter metrics, as evident from the depicted contrast in the percentage of times one region/tract is consistently surviving regularization in a model with only the metrics on one tissue versus a model including the metrics of both gray and white matter. Despite this overlap, certain regions still reliably predict cognitive performance, highlighting the distinct information observed within both tissue types.

### Some metrics are more predictive of cognitive performance than others

**Figure 5.**
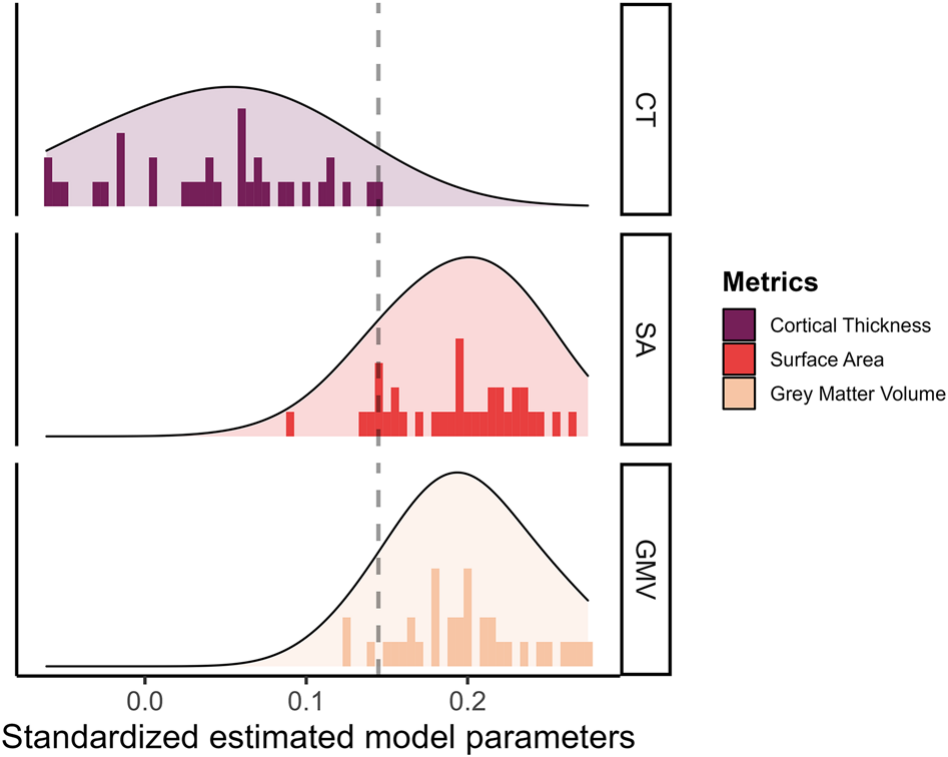
Standardized estimated model parameters on how one region of interest in one metric predicts cognitive performance in the models per ROI per metric for the three gray matter metrics.

Next, we investigated if the different metrics in each tissue have a unique predictive role, and thus if the choice of the metric was important when you want to predict cognitive performance. Comparing the predictive power of the different metrics, we plotted the standardized estimate model parameters of every region across metrics. Figure 5 shows the range of observed values in the path estimates for each metrics, within the gray matter metrics, SA (range $\beta_{\mathrm{std}}$, [0.092;0.265]) and volume (range $\beta_{\mathrm{std}}$, [0.123;0.275]), were overall stronger predictors of cognitive performance compared with those for CT (range $\beta_{\mathrm{std}}$, [−0.06;0.145]). This is somewhat surprising given the prominence of CT as the metric of choice in (developmental) cognitive neuroimaging studies of individual differences. CT is commonly used both in healthy and case–control studies, with five times more papers combining CT and cognition compared with the number of papers using GMV and cognition since 2016 [*N* = 2,351 for CT and *N* = 506 for GMV in a PubMed search as of 30/10/2023: (("cortical thickness"[tiab:∼0])) AND (("cognit*"))].

**Figure 6.**
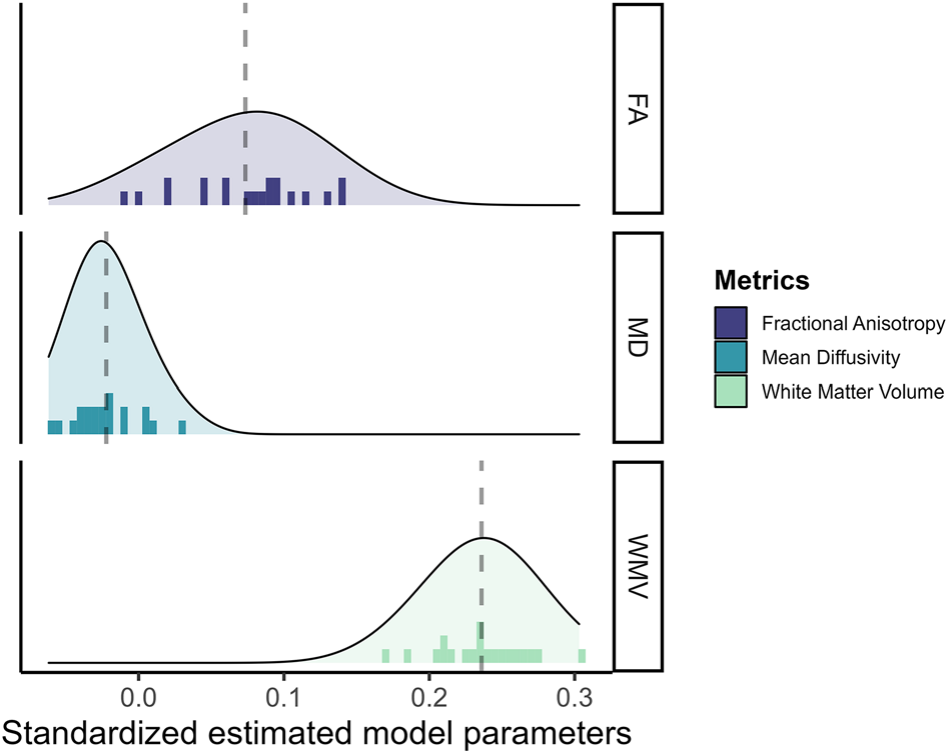
Standardized estimated model parameters on how one region of interest in one metric predicts cognitive performance in the models per ROI per metric for the three white matter metrics.

The results for the white matter metrics is represented in Figure 6. Surprisingly, WMV is overall the strongest predictor of cognitive performance (range$\beta_{\mathrm{std}}$, [0.169;0.303]), followed by FA (range $\beta_{\mathrm{std}}$, [−0.010;0.142]) and MD (range $\beta_{\mathrm{std}}$, [−0.062;0.031]).

These findings were replicated in both male and female participants.

These results provide evidence that different metrics within gray and white matter better predict cognitive performance.

### Within a metric, not every region gives the same information to the prediction of cognitive performance

In addition to different metrics showing distinct predictive strengths as observed above, it is also highly plausible (Basten et al., 2015) that there is regional specificity. In other words, that different regions of interest might have a unique predictive role, and that this role depends on the metrics being studied. To assess the effects of the different regions within a metric, we compared a model with freely estimated parameters to a model where all the parameters are equality constrained, which captures the hypothesis that each region contributes equally. With three exceptions, the free models showed the best fit to the data (AIC_diff_ > 50; BIC_diff_ > 15 in favor of the models with freely estimated parameters for 15/18 of the models with all the regions and the ones with the regularized regions). These exceptions pertained exclusively to BIC criteria in the models with all the regions, and their occurrence can be attributed to the penalization of increased model complexity.

In addition, the regularized models, which favor sparse models with only few predictors, always retained multiple regions even within the same metric. If a single “key” region contains all relevant predictive information, we would not expect to observe this pattern. Across the nine models that underwent regularization, each model estimation showed at least 30% of all regions to have a regularized β different from zero, demonstrating the regional specificity and complementarity hypothesized above.

**Figure 7.**
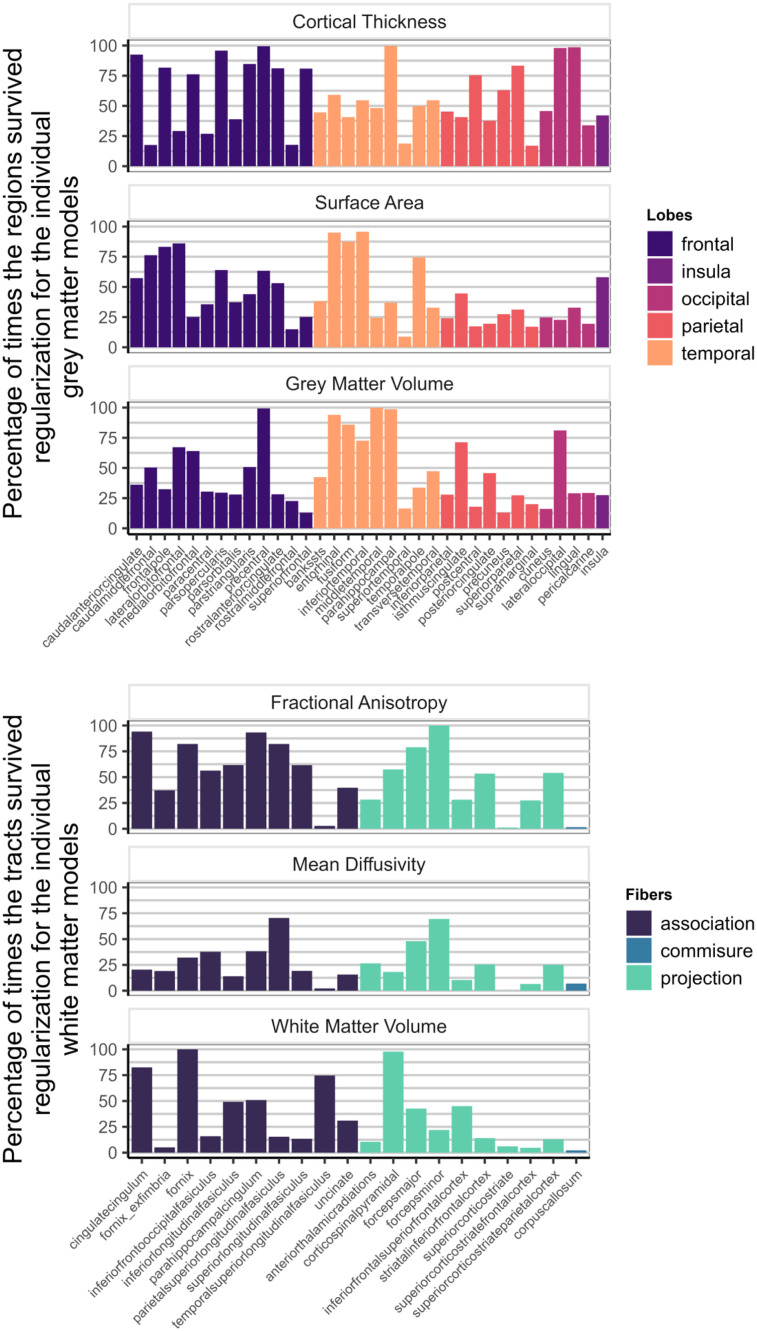
Percentage of times individual region/tract in a metric survived regularization in the individual models for each metric (Fig. 1*D*).

Figure 7 shows how often each region appeared in the final, regularized models across 1,000 different subsets of the data. Regions or tracts with a high percentage of survival are those that most consistently provide unique information to predict differences in cognitive performance compared with all other different regions and tracts within the metric. For instance, a researcher interested in the parietal cortex will see in Figure 7 that regions within this lobe are more consistently important (compared with regions in other lobes) in a model including only CT than in a model that analyzes SA or GMV.

These converging findings demonstrate that to have a better picture of cognitive performance, we would need to assess different regions in a metric simultaneously.

### Across metrics, different regions give unique information to the prediction of cognitive performance

Finally, we investigated if the same regions of interest bring unique information to predict cognitive performance across different metrics in each tissue (e.g., if the FA and white matter in a specific tract contain similar information, then the regularization will likely regularize one of the metrics to 0 for that given tract). A lasso regression was estimated with every region of interest in each gray (or white) matter metrics; the remaining regions were entered in a model as predictors of cognitive performance. This regression allows us to check which regions were nonessential to the prediction of cognitive performance when taking into account all the regions (or tracts) in the three metrics.

**Figure 8.**
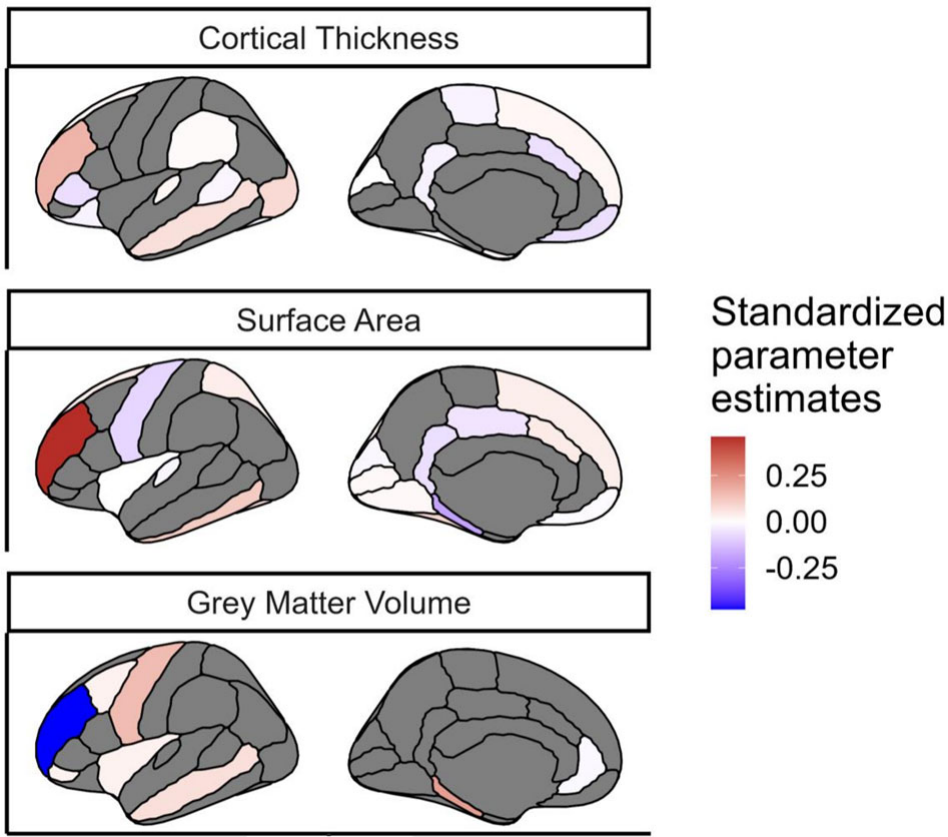
Regions of interest in each gray matter metric that survived the regularization in the model including the three metrics of gray matter. The metrics were averaged bilaterally across the hemispheres. The color indicated the standardized estimated model parameters. The regions in gray did not survived regularization for this model. The regions in white survived regularization but have a standardized estimated model parameter that is close to 0; these regions survived regularization likely because it improved the predictive power of other regions in the model.

We found that despite the strong penalty included in the regularizations, the model still retained regions from each of the gray matter metrics, as well as each of the three white matter metrics, to be significantly predictive of cognitive performance (Fig. 8). This suggests that different regions/tracts give unique information to the prediction of cognitive performance.

**Figure 9.**
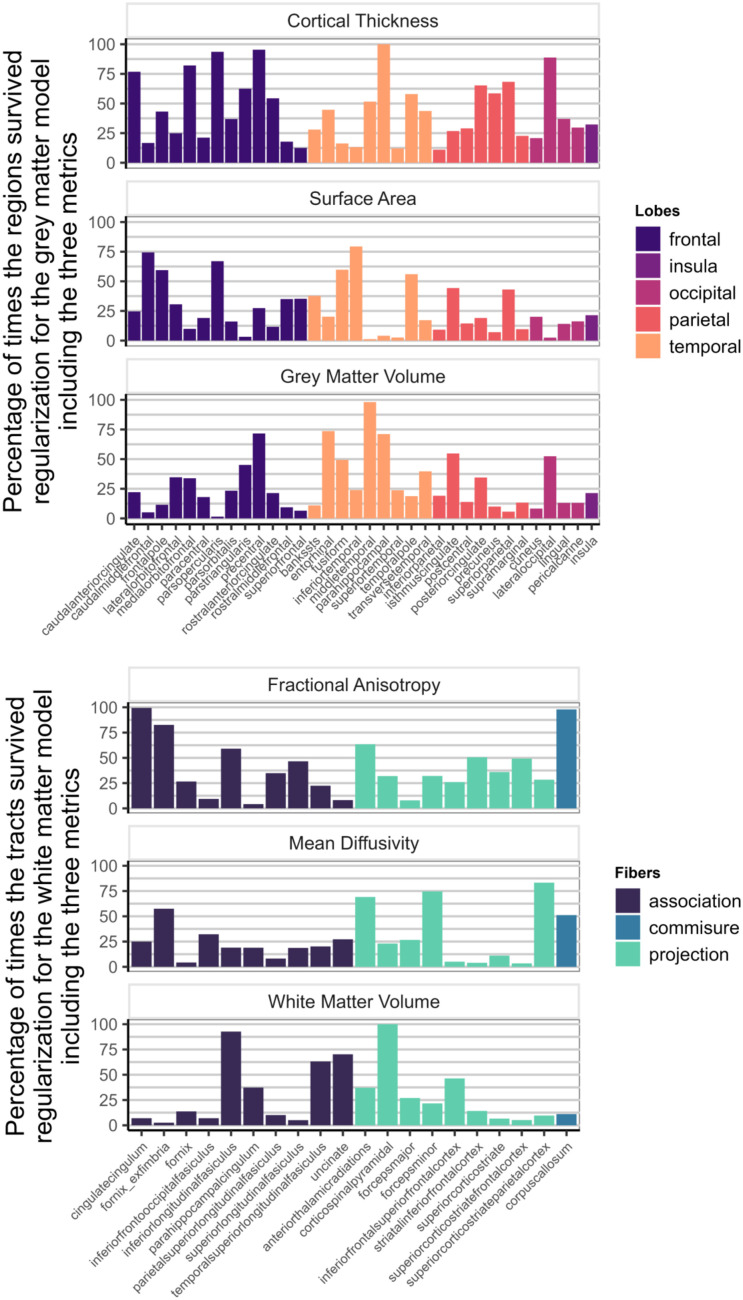
Percentage of times one region in a metric survived regularization in the model with every gray matter metrics included (top figure—Fig. 1*E*) and in the model with every white matter metrics (bottom figure—Fig. 1*F*).

Figure 9 illustrates the survival rates of each region/tract across 1,000 regularizations in a model incorporating either the three gray matter metrics or the three white matter metrics as predictors of cognitive performance. Comparing the percentage values with those in Figure 7, it becomes evident that a portion of the unique information associated with each region in each metric becomes redundant when performing the regularization analysis with combined gray and white matter metrics.

The complete results of all the models studied in this paper are available in OSF (https://osf.io/eygwz).

## Discussion

In a large developmental sample, we examined the role of gray and white matter in supporting cognitive performance. Using regularized structural equation model, we observed that the variance in cognitive performance explained by both gray and white matter (19.0%) is considerably greater than either one in isolation (15.4/12.4%), but not fully additive. This demonstrates that gray and white matter structure bring both unique and shared information in predicting individual differences in cognitive abilities in children.

The observed shared information is unsurprising, given the interdependence of these tissues both during development and aging (Giorgio et al., 2010; Hoagey et al., 2019). Previous work suggests close associations between gray and white matter during development (Tamnes, Østby, Fjell, et al., 2010; Kochunov et al., 2011) and some similarity in the ability of gray and white matter measures to predict cognition (Hoagey et al., 2019). Zhao et al. (2021) proposed that differences in sample ages may explain differences in regional- and metric-specific findings (Zhao et al., 2021) in line with Østby et al. (2011) and Fuhrmann et al. (2020) who observed age-dependent differences in the nature and strength of the relationship between gray matter, white matter, and cognitive performance (Østby et al., 2011; Fuhrmann et al., 2020).

Regarding the secondary goals, we found evidence that different metrics within gray and white matter have distinct predictive power in this sample. For gray matter, SA and volume show the best predictive power compared with CT, a surprising finding given the prevalence of studies which (only) use CT as a structural predictor of cognitive performance. However, this result is consistent with several studies reporting that SA is more related to cognitive performance than CT (Borgeest et al., 2021; Fürtjes et al., 2023; Pulli et al., 2023). Moreover, recent evidence within the same sample suggests that CT has lower reliability than SA or volume, which may differentially attenuate the effects a given study observes (Parsons et al., 2023).

For white matter, we found that in this sample, WMV is the strongest predictor compared with more “advanced” diffusion-based metrics such as FA and MD. This is also unexpected since tract integrity measures are seen as more closely associated with the underlying physiology of tracts and their lesions (Fjell et al., 2008; Xing et al., 2021).

The regional differentiation found at the level of the metrics and the tissues in our models illustrates the importance of selecting regions of interest. Some regions emerge as robust predictors of variance in cognitive performance, while others contribute minimally or lack unique informational value. However, it is apparent that the predictive efficacy of individual regions/tracts varies across the metrics within the models. For instance, CT of the superior frontal region consistently predicts cognitive performance (Fig. 7), but SA or volume of the same region is considerably less predictive. In general, the inclusion of additional metrics within a model is associated with lower frequency of a region/tract's survival through regularization, in line with partially overlapping predictive information across different metrics (Fig. 4). In contrast, and somewhat surprisingly, the inclusion of TIV as a covariate had negligible consequences for both the parameter estimates and the overall explanatory power of the model. Focusing on gender as a potential factor by fitting the same model separately yielded similar overall conclusions, apart from an overall higher predictive performance observed in female compared with that in male. This phenomenon could potentially be explained by variations in motion artifacts (Afacan et al., 2016) or an accelerated cortical maturation process in girls during development (Grabowska, 2017; Fuhrmann et al., 2020, fig 4*c*).

Our study has several strengths. The uniquely large childhood sample of ABCD allows us to both explore the optimal model in an exploratory sample of only 15% of the data and validate it in a test set (Srivastava, 2018). As such the sample size enables us to optimize exploration and confirmation, as well as increase parameter precision and power in line with recent recommendations (Marek et al., 2022). Moreover, by using a previously validated measurement model for the latent cognitive variable, we further decrease measurement error and increase power and precision (Sauce et al., 2022).

However, the main strength of our study is what is commonly absent in the empirical literature: a multimodal analysis including several measures of gray and white matter and the comparison of their explanatory performance. Although previous studies have simultaneously integrated multimodal imaging data (Richard et al., 2020; Rasero et al., 2021), those more commonly examine the individual covariances between multimodal components or factors rather than examine whether distinct metrics provide unique complementary predictive information. The challenge in models with many predictors is that additional predictors will always increase the strength of the joint prediction, at least within a sample. In our modeling approach, we rely on three approaches to guard against needless complexity to differentiate the three models: (1) regularization penalizes the predictive estimates downward to ensure a parsimonious (and often sparse) model; (2) model comparison criteria that penalize unnecessary complexity, favoring a model with fewer predictors that does “almost” as well as the more complex model (Jacobucci et al., 2019); and (3) report effect sizes as adjusted *R*-squared to account for the number of parameters needed to achieve an overall amount of variance explained.

Despite these strengths, our study also has several limitations. First and foremost, the present analysis focuses on cross-sectional differences, in a specific age range (9–11 years old). This is a unique developmental period for the brain during which gray matter starts to decrease, while white matter continues to increase (Bethlehem et al., 2022), so we expect the precise pattern of brain–behavior relationship to be contingent on this specific developmental period. Future studies will extend our results to a longitudinal approach to tease apart leading and lagging effects and provide additional support in line with causal hypotheses (King et al., 2018; Vijayakumar et al., 2018). Moreover, although ABCD undertook considerable efforts to recruit a representative sample of the USA, our findings cannot be assumed to generalize to more diverse populations within and especially beyond the USA (LeWinn et al., 2017; Garavan et al., 2018).

In terms of methodology, the ABCD sample has been collected across different sites and scanners, which may include site or scanner variance we did not incorporate [although Parsons et al. (2023) suggest that these site effects in ABCD are considerably smaller than other sources of (un)reliability]. Moreover, our findings will be affected by certain pragmatic methodological choices. For example, bilaterally averaging the regions of interest across the hemispheres allowed the possibility to do both regularization and maximum likelihood with a large number of regions. However, it precludes us observing any hemispheric specificity on the prediction of cognitive performance. Finally, the definition of gray and white matter as two different tissues measured by different metrics is inherently an oversimplification. MRI-derived measures are proxies of the underlying brain structure and are not able (yet) to isolate specific biophysiological components of gray or white matter. For instance, (apparent) CT is known to be sensitive to myelinization of adjacent white matter (Natu et al., 2019), suggesting that a partial overlap in statistical contributions of gray and white matter may be as much methodological artifact as biological reality. Finally, segmenting the brain into defined regions reduced structural complexity, correspondingly diminishing explained cognitive performance variance (Fürtjes et al., 2023).

To develop a full picture of the complementary roles of gray and white matter to the prediction of cognitive performance, future studies will need to examine large, multimodal, longitudinal, and crucially diverse samples across the life span. In addition, numerous new measures are needed to estimate cellular mechanisms more accurately. For instance, sulcal depth and curvature analysis provides detailed cortical structure mapping, while myelin water fraction or magnetization transfer ratio offers more direct approaches to study axons’ microstructure and myelinization (Timmers et al., 2016; Lipp et al., 2019). Future work will benefit from metrics that demonstrate a better representation of underlying cellular mechanisms (Goriounova and Mansvelder, 2019). Beyond the metrics, the emergence of new scanners with higher resolution like the 11.7 T or the CONNECTOM scanners will help unveil the structure within the six layers of the cortex and regional corticocortical connectivity (Nowogrodzki, 2018; Huang et al., 2021; Raven et al., 2023).

The present study was designed to evaluate the extent of the overlap between gray and white matter metrics in the prediction of cognitive performance. Our findings suggest that studies focusing solely on one tissue or one metric when linking brain and cognition are likely missing out on complementary explanatory power. Studies limited by pragmatic concerns should carefully consider which metric to focus on, informed by the phenotype of interest and the population being studied, and make explicit that the findings are likely contingent on the metrics used in a study. Future work should incorporate a more holistic view of brain structure across modalities, metrics, and measures to better elucidate the relationships between brain and cognitive performance.
